# *Dirofilaria immitis* in dogs from the coastal tourist region of the state of Alagoas, Brazil

**DOI:** 10.1590/S1984-29612024055

**Published:** 2024-09-16

**Authors:** Walter Franklin Bernardino Leão, Viviane Melo Coelho Barros, Eduarda Viana Mafra Cardoso, Flávia Silva Damasceno, Juaci Vitória Malaquias, David Germano Gonçalves Schwarz, Abelardo Silva-Júnior, Wagnner José Nascimento Porto

**Affiliations:** 1 Universidade Federal de Alagoas – UFAL, Maceió, AL, Brasil; 2 Programa de Pós-graduação em Ciência Animal, Laboratório de Parasitologia, Instituto de Ciências Biológicas e da Saúde, Universidade Federal de Alagoas – UFAL, Maceió, AL, Brasil; 3 Embrapa Cerrados, Planaltina, DF, Brasil; 4 Centro de Ciências Agroveterinárias, Universidade do Estado de Santa Catarina – UDESC, Florianópolis, SC, Brasil

**Keywords:** Northeastern Brazil, dirofilariasis, heartworm, dogs, Nordeste brasileiro, dirofilariose, verme do coração, cães

## Abstract

Canine dirofilariasis, caused by *Dirofilaria immitis*, is prevalent worldwide. However, the frequency of canine infection in the state of Alagoas, Brazil is scarcely unknown. This study aimed to evaluate the frequency of *D. immitis* infection in dogs from the coastal municipalities of Alagoas and analyze the risk factors associated with the infection. A cross-sectional study was performed using 426 dogs of different breeds and sex distributed across 15 coastal municipalities in the state of Alagoas. Blood samples were collected from each dog and analyzed for circulating microfilariae and free *D. immitis* antigens. To investigate the risk factors associated with *D. immitis* infection, we collected information on dog environments using an epidemiological questionnaire. The results revealed that 12.7% of dogs tested positive for *D. immitis*. Dogs with travel history were 3.52 times more likely to be infected. Thus, infected dogs in the coastal region of Alagoas should be regularly monitored and the public health system should plan strategies to control this zoonotic disease.

## Resumo

A dirofilariose canina é uma enfermidade de distribuição mundial, causada por *Dirofilaria immitis*. No estado de Alagoas, pouco se sabe sobre a frequência da infecção por *D. immitis* em cães. Assim, o objetivo deste estudo foi avaliar a frequência da infecção por *D. immitis*, em cães dos municípios da região litorânea de Alagoas, como também analisar os fatores de risco para a infecção. Para isso, foi realizado um estudo transversal, com uma população amostral de 426 cães, de diferentes raças e sexo, distribuídos em 15 municípios litorâneos. Amostras de sangue foram coletadas de cada um dos cães e analisada a presença de microfilárias circulantes e antígenos de *D. immitis*. Para avaliar os fatores de risco, foi utilizado um questionário epidemiológico com perguntas relacionadas ao animal e ao ambiente. No total, 12,7% dos cães estavam infectados por *D. immitis*. Verificou-se também que os cães com histórico de viagens, apresentaram 3,52 vezes mais risco de serem positivos. Dessa forma, a presença de cães infectados na região estudada demonstra a necessidade de vigilância e controle por parte do sistema público de saúde, visto que é uma doença com potencial zoonótico.

Canine dirofilariasis, also known as heartworm disease, is caused by the nematode, *Dirofilaria immitis*. Although dirofilariasis is prevalent worldwide, endemic areas include regions with tropical and temperate climates that are favorable for vector growth. The main transmission vectors of dirofilariasis are mosquitoes belonging to different genera such as *Aedes*, *Anopheles*, *Culex*, and *Ochlerotatus* (Otranto et al., 2013).

The incidence of the infection is higher in coastal regions with warm climates; however, dirofilariasis cases have also been reported in non-coastal regions (Simón et al., 2012; Dantas-Torres & Otranto, 2020; Labarthe et al., 2014; Soares et al., 2022). Similar to dogs, cats and humans can be infected with *D. immitis* via mosquito bites (Campos et al., 1997; Mendes-de-Almeida et al., 2021).

Several factors can interfere with canine *D. immitis* infection prevalence rates, such as the vector population, unprotected canine population, climate, number of microfilaremic dogs, age, handling, and travel to endemic regions. Therefore, vector behavior, their adaptation to new environments, and the transport of dogs to different regions are important factors behind the worldwide distribution of the infection (Labarthe et al., 2014; Morchón et al., 2012).

However, information regarding the frequency and distribution of canine heartworm infection in the state of Alagoas in Brazil is limited. A 20-year-old study investigated the prevalence of the microfilaremic canine *D. immitis* infection in the municipality of Maceió, the capital of the state of Alagoas, and reported a prevalence of 3.1% (Brito et al., 2000). The coastal region of Alagoas is a popular destination for tourists from various parts of Brazil and abroad. During 2002 more than two million people visited the State (Alagoas, 2023). The aim of this study was to evaluate canine heartworm infection frequency in all coastal municipalities of Alagoas and analyze the risk factors associated with infection.

The state of Alagoas is located in the north-eastern region of Brazil and shares borders with the states of Pernambuco (north and northwest), Sergipe (south), Bahia (southwest), and the Atlantic Ocean (east). The state has a population of approximately 3.365,351 inhabitants and a population density of 112.33 inhabitants/km^2^. The state has 102 municipalities; of these, 15 are coastal, including the capital, Maceió. Alagoas has a tropical humid climate, with temperature average of 26.14 °C, rainfall of approximately 1.304 mm annually, and a humidity of 77.33% (Climate Data, 2023).

A cross-sectional study was conducted to investigate the risk factors associated with the frequency of *D. immitis* infection in 15 coastal municipalities of the state of Alagoas, Brazil. From August 2016 to November 2018, 426 dogs six months or older (197 females and 229 males) domiciled and semi-domiciled were sampled. For sample collection, the dogs were physically contained by their owners, and 5 ml of venous blood were drawn and transferred to tubes with or without anticoagulants.

The blood samples were kept at approximately 4^o^C until processing at the laboratory, no longer than five days. Serum was obtained from the blood samples by centrifugation at 2000 × *g* for 10 min, and the samples were stored at -20 °C until further analysis. For parasitological diagnosis, a modified Knott’s technique (Knott, 1939; Newton & Wright, 1956) was used to investigate circulating microfilariae in whole blood samples. The samples were also screened using Snap®4Dx® Kit (IDEXX Laboratories, Westbrook, USA) according to manufacturer’s instructions. The dogs were considered *D. immitis* infected when positive in both techniques.

A pre-structured epidemiological questionnaire was used to collecting information related to the animals and their environment (sex, age, clinical signs, breed, dog size, coat length, coat color, deworming, presence of mosquitoes, travel history and nocturnal habits). Questionnaires were designed for dichotomous responses and tabulated or systematized using Microsoft Excel 2013 software spreadsheets.

Data were analyzed using absolute and relative frequencies. Association analyses were performed between municipalities with positive cases using Fisher’s exact test. To assess the bivariate association between the dependent variable (infection) and the independent variables, Pearson’s chi-square test and Fisher’s exact test were used to analyze the data considering a confidence interval of 95% and P ≤ 0.05. For multivariate analysis, all variables were included in the analysis, regardless of the level of significance in the bivariate analysis. For that, nominal logistic regression model was used to identify multivariate associations between the dependent variable and the independent variables, with a margin of error of 5%, in the statistical program R package version 4.2.1.

**Figure 1:**
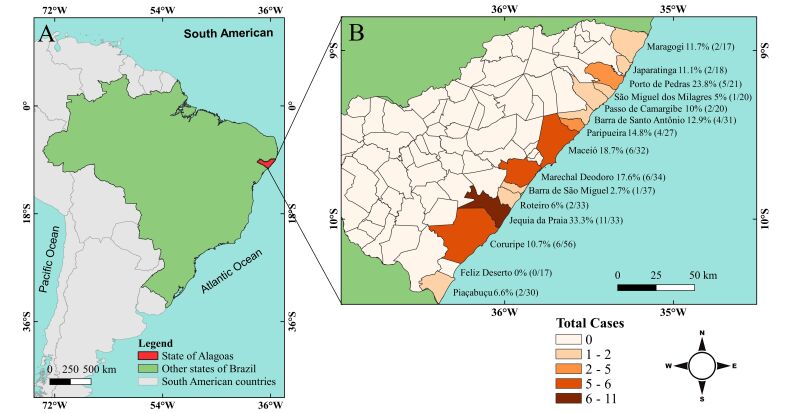
Map of the area investigated in this study. (A) Map of Brazil, highlighting the state of Alagoas in red; (B) Map of the state of Alagoas, highlighting the municipalities in the coastal region, showing the absolute numbers of dogs tested and those that were positive for *Dirofilaria immitis*.

The result showed that 12.7% (54/426) of the dogs tested positive for *D. immitis* infection. Moreover, it is worth mentioning that the parasite circulates in 93% (14/15) of the coastal municipalities of the state (Figure 1B). Jequiá da Praia presented the highest frequency of infected dogs (33.3%; 11/33), followed by Porto de Pedras (23.8%; 5/21) and Maceió (18.8%; 6/32), (Figure 1B).

Dogs with travel history were 3.52 times more likely to be infected (Table 1).

**Table 1:** Bivariate and multivariate analysis between heartworm infection and associated risk factors in domiciled and semi-domiciled dogs from the coastal region of the state of Alagoas, Brazil.

	Positive		Negative		Total	HTW-ag		Multivariate	
Risk factors						Bivariate			
	n	(%)	n	(%)		*p*-value	OR (CI 95%)	*p*-value	OR (CI 95%)
Coast region						1.000	1.05 (0.57;1.88)	0.97	1.01 (0.51;2.02)
North coast	20	4.7	134	31.5	154				
South coast	34	8.0	238	55.9	272				
Breed						0.8821	0.87 (0.34;1.92)	0.83	1.11(0.41;3.02)
Yes	7	1.6	55	12.9	62				
No	47	11.0	317	74.4	364				
Gender						0.01335*	0.45 (0.23;0.82)	0.07	1.87(0.95;3.66)
Female	16	3.8	181	42.5	197				
Male	38	8.9	191	44.8	229				
Big Size						0.5264	1.56 (0.55;3.75)	0.58	0.74(0.26;2.14)
Yes	6	1.4	28	6.6	34				
No	48	11.3	343	80.5	391				
Short Coat						0.2369	1.57 (0.82;3.24)	0.15	0.57(0.27;1.22)
Yes	41	9.6	254	59.6	295				
No	12	2.8	118	27.7	130				
Dark color						0.9333	1.08 (0.58;2.10)	0.92	1.04(0.52;2.07)
Yes	15	3.5	107	25.1	122				
No	39	9.2	257	60.3	296				
Clinical signs						0.0030*	0.35 (0.18;0.70)	0.01*	2.66(1.28;5.52)
Yes	15	3.5	44	10.3	59				
No	39	9.2	328	77.0	367				
Deworming						0.8319	1.11 (0.61;2.06)	0.76	0.90(0.46;1.77)
No	19	4.5	143	33.6	162				
Yes	34	8.0	229	53.8	263				
Presence of mosquitoes						0.6754	1.40 (0.50;3.34)	0.94	0.96(0.33;2.76)
Yes	48	11.3	341	80.0	389				
No	6	1.4	31	7.3	37				
Travel history						0.4251	0.59 (0.24;1.68)	0.03*	3.52(1.14;10.92)
Yes	6	1.4	26	6.1	32				
No	48	11.3	346	81.2	394				
Guard role						0.0824	0.53 (0.28;1.05)	0.34	1.45(0.67;3.13)
Yes	15	3.5	3	0.7	18				
No	39	9.2	309	72.5	348				
Nocturnal habits in the backyard						0.05*	0.38 (0.13;0.91)	0.06	2.89(0.96;8.67)
Yes	49	11.5	292	68.5	341				
No	5	1.2	80	18.8	85				

HTW-Ag = *Dirofilaria immitis* antigen; OR = odds ratio; CI = confidence interval.

To the best of our knowledge, this is the first study evaluating the frequency of *D. immitis* infection in dogs along the coast of Alagoas.

In Maceió the reported frequency of infected microfilaremic dogs, according to microfilariae detection ranged from 1.3% to 3.1% (Brito et al., 2000, 2001). The frequency of the cases described in the present study show an important increase when compared with those reported before (Brito et al., 2000, 2001). Although in the present study antigen detection was used, it must be highlighted that all the 54 infected dogs presented in this survey were microfilaremic as well.

The increase of infected dogs can be attributed to environmental factors. Environmental changes caused by anthropogenic activities, such as clima changes may facilitate the proliferation and maintenance of mosquito populations. Extrinsic temperature rise accelerates larvae development in the mosquitoes at the same time it also increases the mosquitoes development (Morchón et al., 2012). Furthermore, since it is a touristic pet friendly environment, the increased frequency of microfilaremic dogs may contribute with the spread of the parasite in unestimated proportions.

Risk factors for *D. immitis* infection include the physiological state of the animal, exposure to vectors and transport to endemic regions (Perez-Sanchez et al., 1989; Tzipory et al., 2010). Environmental factors are also closely linked to the transmission of *D. immitis*, such as rainfall, relative humidity, vegetation indices, population density of dogs and vectors, and socioeconomic factors (Brown et al., 2012). In this study, the risk factors for canine heartworm infection, including sex, size, coat length, coat color, deworming, travel history, nocturnal habits, and presence of mosquitoes in the domicile were studied. Although previous reports showed that travelling did not increase risk (Labarthe et al., 2014; Trancoso et al., 2020; Barbosa et al., 2023), in the present study when travel history was tested, it was shown that travel played an important role. Travelling dogs were more susceptible to *D. immitis* infection than non-travelling dogs (Table 1).

The presence of heartworm disease along the entire coast of Alagoas (except in one municipality, which however is located between two municipalities with infected dogs) has raised concerns for public health in the state because *D. immitis* can also affect humans. Cases of heartworm disease in humans have been reported worldwide (Otranto et al., 2013, Bublitz et al., 2012; Dantas-Torres & Otranto, 2014). In humans, the parasite does not reach the adult stage, giving rise to pulmonary nodules that can be misdiagnosed as a neoplasm (Campos et al., 1997).

This study describes for the first time the presence of *D. immitis* infection in dogs along the tourist coast of Alagoas. The presence of infected dogs in the 93.33% of the coastal region demonstrates the need for surveillance and control by the public health system, as this infection can also affect residents and tourists visiting the region.
